# SIRT1 Promotes Neuronal Fortification in Neurodegenerative Diseases through Attenuation of Pathological Hallmarks and Enhancement of Cellular Lifespan

**DOI:** 10.2174/1570159X18666200729111744

**Published:** 2020-07-29

**Authors:** Priya Mishra, Amit Kumar Mittal, Harikesh Kalonia, Swati Madan, Shampa Ghosh, Jitendra Kumar Sinha, Satyendra Kumar Rajput

**Affiliations:** 1 Amity Institute of Neuropsychology and Neurosciences (AINN), Amity University, Noida, Uttar Pradesh 201313, India;; 2 Amity Institute of Indian System of Medicine (AIISM), Amity University, Noida, Uttar Pradesh 201313, India;; 3 Amity Institute of Pharmacy (AIP), Amity University, Noida, Uttar Pradesh 201313, India;; 4 ICMR – National Institute of Nutrition, Tarnaka, Hyderabad, Telangana 500007, India

**Keywords:** SIRT1, neurogenesis, genomic stability, oxidative stress, neuoroprotection, neurodegenerative disease

## Abstract

Neurodegeneration is a complex neurological phenomenon characterized by disturbed coherence in neuronal efflux. Progressive neuronal loss and brain damage due to various age-related pathological hallmarks perturb the behavioral balance and quality of life. Sirtuins have been widely investigated for their neuroprotective role, with SIRT1 being the most contemplated member of the family. SIRT1 exhibits significant capabilities to enhance neurogenesis and cellular lifespan by regulating various pathways, which makes it an exciting therapeutic target to inhibit neurodegenerative disease progression. SIRT1 mediated neuronal fortification involves modulation of molecular co-factors and biochemical pathways responsible for the induction and sustenance of pro-inflammatory and pro-oxidative environment in the cellular *milieu*. In this review, we present the major role played by SIRT1 in maintaining cellular strength through the regulation of genomic stability, neuronal growth, energy metabolism, oxidative stress, inhibiting mechanisms and anti-inflammatory responses. The therapeutic significance of SIRT1 has been put into perspective through a comprehensive discussion about its ameliorating potential against neurodegenerative stimuli in a variety of diseases that characteristically impair cognition, memory and motor coordination. This review enhances the acquaintance concerned with the neuroprotective potential of SIRT1 and thus promotes the development of novel SIRT1 regulating therapeutic agents and strategies.

## 1 INTRODUCTION

**Fig. (1):**
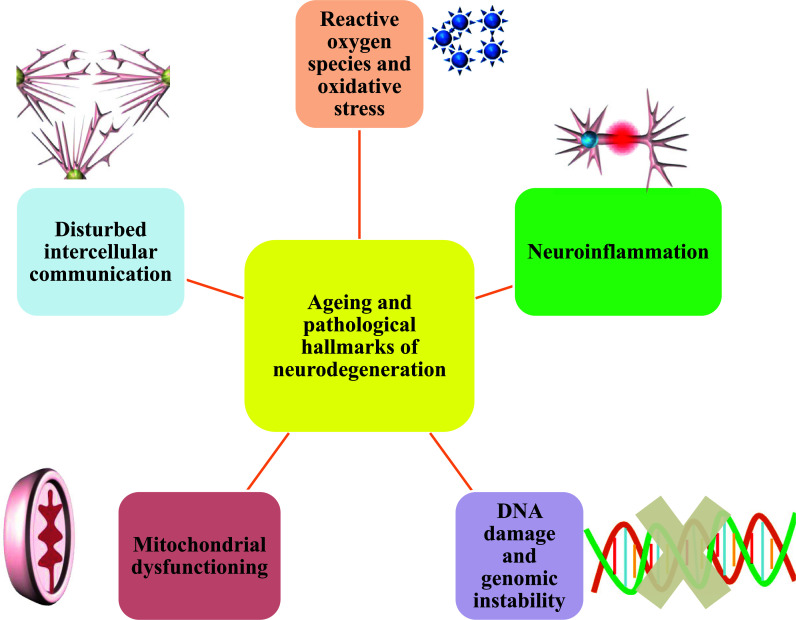
Ageing is one of the most prominent factors of neurodegeneration. Age related neurodegeneration progresses due to a fatal cause-effect loop. Aged brain has significantly diminished capability of DNA repair. The resulting genomic instability deteriorates intercellular communication, thereby affecting the normal functioning of the neuronal circuits. It also enhances inflammation in the neurons which is further facilitated by reduced autophagy and mitophagy. While reduced autophagy results in the accumulation of toxic misfolded proteins and inflammation promoters, the hindrance in mitochondrial functioning caused due to reduced mitophagy renders reactive oxygen species unchecked that elevates oxidative stress. These free radicals further damage the DNA and hamper mitochondrial biogenesis and fuel the cause-effect loop of neurodegeneration. Such progressive and continuous neuronal loss overcomes the natural process of neurogenesis and thereby leads to irreversible brain damage and subsequent onset of neurodegenerative diseases.

Neurodegeneration refers to the progressive brain damage leading to fatal disruption of the neuronal circuits. Progressive atrophy in neurodegeneration compromises neuronal functioning and structure. The communication channel within the neuronal *milieu* is ruptured due to large-scale neuronal death, and this eventually jeopardizes cognition, memory, and limbic reflex. The quality of life of the patient is further deteriorated because of the multifaceted nature of neurodegeneration. Exact causative factors are hard to determine and remain elusive in most of the cases; however, ageing is one of the most prominent risk factors agreed upon by the experts. The most common neurodegenerative diseases like Alzheimer’s disease (AD) and Parkinson’s disease (PD) are most commonly found in elderly people and the vulnerability enhances with increasing age. Age related molecular and biochemical changes in the brain create a fatal loop of pathological hallmarks (Fig. **1**) that progressively damages different brain parts. Age dependent neurodegeneration not only leads to the burden of lost lives but also gives a considerable jolt to the local and global economy. The need for sophisticated health care facilities, patient care shelters, and trained personnel for the treatment and care of patients makes the monetary figures increasingly astounding. It is estimated that USA alone would be spending around 1.1 trillion dollars per annum on the treatment and care of the AD patients [1]. Therefore, it is imperative to investigate and find potential molecular targets within the brain parenchyma whose regulation can be used for achieving desired therapeutic potency against neurodegenerative disorders.

Modulation of mammalian sirtuins presents an efficient therapeutic strategy against neurodegenerative disorders. The sirtuins (SIRT1-7) are class III histone deacetylases (HDACs), which are one of the most broadly conserved proteins from bacteria to humans. Sirtuins regulate the acetylation of several histone and non-histone proteins, thereby exhibiting complex functions. They depend upon NAD^+^ (nicotinamide adenine dinucleotide) for activation [2-5]. While each member of the sirtuin family has specific importance, SIRT1 holds a significantly vital place in the domain of neuropathology of various disorders related to the central nervous system (CNS) [6-8]. It is certainly the most extensively contemplated member of the sirtuin family. The neurotherapeutic implication of SIRT1 is attributed to its attenuating potential against pathological markers of neurodegeneration. SIRT1 regulation facilitates smooth physiological processes, thereby increasing cell life and inhibiting the progression of neurodegenerative stimuli in the brain. Hence, the study of its regulatory mechanisms and pathways can potentiate the development of specific therapeutic agents and strategies against a variety of neurodegenerative disorders.

## 2 PATHOLOGICAL HALLMARKS OF NEURODEGENERATION

The ageing process enhances neuronal death and subsequent brain damage by facilitating the development of pathological hallmarks of neurodegeneration (Fig. **1**). Ageing related neuronal death is promoted due to oxidative stress mediated DNA damage that subsequently compromises the genomic stability and intercellular communication in the brain [9, 10]. Genomic instability acts as both the cause and effect of ageing related complexities through enhanced mutations and epimutations that render the genome maintenance system ineffective, which thereby hampers the cellular ability to cope with chemical lesions. This pervades signaling cascades throughout the cellular environment and depletes NAD^+^, resulting in downregulated DNA repair mechanisms and mitophagy [11, 12]. Mitophagy is essential to get rid of the degraded mitochondria through the process of autophagy to promote cellular quality control and reinstate homeostasis in the cellular parenchyma. Such natural detoxifying and cleansing mechanisms prevent the onset of a myriad of neurological abnormalities by facilitating for balanced equilibrium and diminished concentration of toxins in the cellular *milieu*. Moreover, the greater concentration of aggregates of toxic misfolded proteins upregulates the secretion of inflammatory cytokines and that is responsible for neuroinflammation [13, 14]. Retarded autophagy in the aged brain further enhances the inflammation promoters and facilitates the progression of neurodegenerative stimuli. Furthermore, mitochondrial damage causes energy imbalance and increases in the concentration of reactive oxygen species (ROS) that further weakens the DNA repair system, leading to significant neuroinflammation, which further escalates the neurodegenerative loss in the brain [15-17]. The pro-inflammatory environment gets a boost with increasing age, thereby subsequently compromising the CNS activity through hindered neuronal activity and elevated concentration of inflammatory cytokines. Such progressive alterations contribute towards cognitive decline and therefore deteriorate the quality of life.

## 3 SIRT1 FACILITATES CONTROL OVER VITAL BRAIN FUNCTIONS

**Fig. (2):**
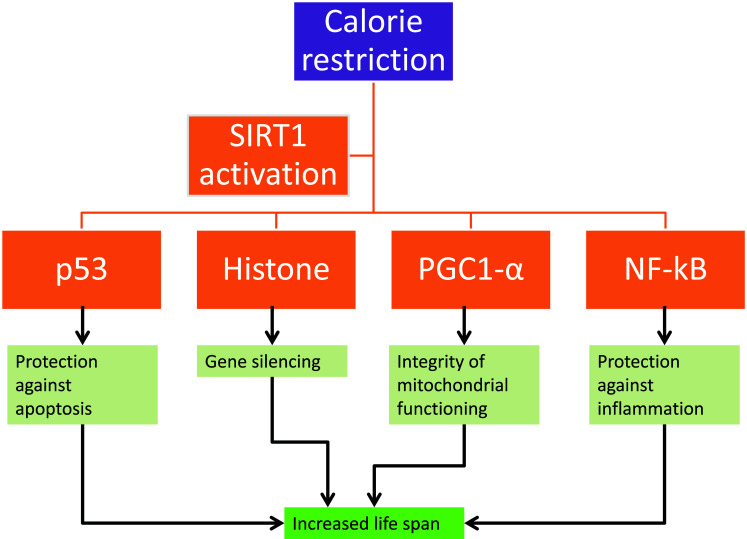
The calorie restriction (CR) process is known to activate the SIRT1 molecule that leads to the regulation of various genes and proteins. SIRT1 regulates the p53 unit and provides substantial protection against cell apoptosis, which is one of the leading causes of cell-death. The histone deacetylation potential of the SIRT1 molecule results in increased life span through gene silencing. SIRT1 is also responsible for the regulation of PGC1-α, which is responsible for up-keeping mitochondrial integration and functioning. Energy failure and imbalance in mitochondrial functioning potentiate cellular apoptosis. Neuroinflammation is another important aspect in neuropathology. SIRT1 is responsible for the downregulation of NF-kB activity that has been found to lower neuroinflammation and oxidative stress.

SIRT1 expression is an important modulator of neurodegenerative disease progression. Animal models, especially rodent models, have confirmed the presence of SIRT1 during embryogenesis in the brain, spinal cord, and dorsal root ganglion [18]. SIRT1 is significantly expressed in vital regions of the brain, including the hippocampus, cerebellum, and hypothalamus [18-21]. SIRT1 expression in brain regions that control vital functions of cognition, memory, and motor coordination is of considerable functional importance. Furthermore, high fat diet lowers SIRT1 expression, while physical exercise and diet restriction enhance its expression (Fig. **2**) [22, 23]. This links SIRT1 expression directly with nutritional quality and physiological processes concerned with cellular metabolism.

## 4 SIRT1 REGULATES PHYSIOLOGICAL PROCESSES TO ENHANCE THE CELLULAR LIFESPAN

### 4.1 SIRT1 Regulates Neuronal Growth and Differentiation

The abundant expression of SIRT1 in brain regions bestows it the ability to play crucial roles of therapeutic significance during neurogenesis. SIRT1 downregulation in mice has been shown to impair embryogenesis, signifying its positive effect on neuronal progenitor cells. SIRT1 molecule regulates Sox-2 and Oct-4 [5, 24], thereby facilitating for pluripotency, which is vital for neuronal survival and genesis. However, optimum expression is important because overexpression of SIRT1 is known to retard neurogenesis [25]. SIRT1 shows localization in neural precursor cells and enhances their differentiation by regulating the level and activity of pro-neuronal transcription factor Mash-1 under oxidative stress stimuli [26]. SIRT1 activation and increased NAD/NADH (nicotinamide adenine dinucleotide (NAD)^+^ hydrogen) ratio have been shown to upregulate neural precursor cell differentiation towards the astrocytic lineage [27]. On the other hand, SIRT1 silencing has been shown to promote neuronal production in sub-ventricular zone (SVZ) and hippocampus [28, 29]. The acetylation state of Pax3 mediated through SIRT1 regulation drives the differentiation of sensory neurons. SIRT1 silencing acetylates Pax3 and thereby enhances neuronal production [30]. SIRT1 knockdown through a lentiviral regulated delivery approach enhances neurogenesis in the hippocampus and SVZ, while it has not been found to have any effect on the proliferation of neural precursors [5]. Stem cell specific knockout of SIRT1 enhances the self-rejuvenating rate of adult neural stem cells [31]. Neurogenesis in the adult brain is also enhanced through genetic ablation of SIRT1 [31].

Importantly, SIRT1 activity also regulates neuritogenesis, *i.e.*, the outgrowth of axons and dendrites. SIRT1 increases axonal length and dendritic branching in hippocampal neurons [32]. While SIRT1 modulated Akt deacetylation reduces the activity of glycogen synthase kinase 3 (GSK3) [32], reduced SIRT1 expression increases mTOR signaling and impairs neurite outgrowth [33]. On the contrary, activation by SIRT1 promoters retards this impairment. SIRT1 enhancement through resveratrol is known to increase dendritic branching by regulating Rho-Kinase activity [34]. Additionally, neurite outgrowth is also promoted through SIRT1 mediated deacetylation of p53 protein [35], which is a promoter of neuronal apoptosis. SIRT1 prevents DNA damage by diminishing p53 induced neuronal apoptosis in the cortical region [36, 37]. These results signify the vital influence that SIRT1 has on neurogenesis and neuritogenesis *via* various targets and mechanisms.

### 4.2 SIRT1 Regulation Attenuates Cognition and Memory Impairment

SIRT1 is well known as an antagonist of neurodegenerative processes and thereby inhibits cognition and memory impairment. SIRT1 deficiency in mice significantly impairs short term and long-term memory accompanied by faults in synaptic plasticity [38]. SIRT1 knockout reduces dendritic density in CA1 neurons [39]. SIRT1 suppresses miR-134 and promotes brain-derived neurotrophic factor (BDNF) expression to regulate synaptic plasticity and memory [40]. BDNF is an important regulator of neuronal growth and synaptic plasticity in various brain regions, and its deficiency escalates into neuroapoptosis [40, 41]. Studies show SIRT1 activation by resveratrol negates sevoflurane-induced neurotoxicity through enhanced BDNF expression in young mice brain [42].

SIRT1 plays an important role in the maintenance of genomic stability in the brain and regulates a cascade of events for proper cognitive and learning functions. SIRT1 enhances insulin-like growth factor 1 (IGF1) signaling that eventually affects important functions like learning and memory [43]. The IGF1 gene acts through extracellular signal-regulated kinase (ERK1/2) and mitogen-activated protein kinase (MAPK). Reduction in IGF1 gene causes a decline in ERK1/2, MAPK, and PI3 (Phosphoinositide 3) kinases that are necessary and essential for several brain tasks [44]. In a mice study, it was found that SIRT1 was responsible for enhanced acetylation and diminished activation of ERK1/2 pathway [45], and the whole process was eventually related to oxidative stress resistance. SIRT1 induced deficiency of ERK1/2-MAPK pathway in conjunction with redefined gene expression in synaptic functions is amongst the many mechanisms through which SIRT1 exercises control over vital cognitive functions of learning, memory and synaptic plasticity in the hippocampal region [46].

### 4.3 SIRT1 is Vital for Upholding Energy Metabolism

SIRT1 plays a significant role in metabolic homeostasis. SIRT1 silencing in rat reduced nutrient intake through Forkhead box protein O 1 (FoxO1) regulated enhancement of anorexigenic pro-opiomelanocortin (POMC) and the reduction of orexigenic agouti-related peptide (AgRP) [47]. SIRT1 inhibition in obese rats increases POMC and α-melanocyte-stimulating hormone (α-MSH) that are responsible for enhancing T3 level and thyroid releasing hormones [48]. Overexpressed SIRT1 in POMC and AgRP neurons retards age related weight gain through enhanced energy usage and lowered diet intake [49]. Hence, it plays a vital role in glucose and lipid dependent energy metabolism in tissues, which holds significance in motor functions. SIRT1 induces hepatic gluconeogenesis and enhances muscle insulin sensitivity [50, 51]. Insulin sensitivity is reversed due to SIRT1 silencing. Hence, SIRT1 activity in the hypothalamus mimics the effects of diet restriction, which is well known to promote neuronal survival and cellular lifespan (Fig. **2**) [52]. SIRT1 also regulates energy metabolism in the liver, adipose tissues and skeletal muscle through repression of Peroxisome Proliferator-Activated Receptor gamma (PPARγ) and deacetylation of PPARγ coactivator-1α (PGC-1α) [53, 54]. SIRT1 activates AMP-dependent kinase (adenosine monophosphate-activated protein kinase; AMPK), which is a key modulator of cellular metabolism and thus enhances cellular lifespan [55].

### 4.4 SIRT1 Stimulates Mitochondrial Biogenesis

SIRT1 and PGC-1α ensure the correct cellular metabolism and mitochondrial health. Both are present in the mitochondrial space of platelets and human cell lines. SIRT1 and PGC-1α affect mitochondrial transcription through mitochondrial DNA (mtDNA) and mitochondrial transcription factor A (TFAM) [56, 57]. It is well known that mtDNA encoded genes play a significant role in mitochondrial biogenesis. SIRT1 activation upregulates PGC-1α and subsequently regulates the transcription of TFAM, which in turn stimulates mtDNA replication [58]. PGC-1α activation also promotes transcription of Nuclear respiratory factors (NRF1 and 2) that eventually enhances TFAM transcription and facilitates for the whole replication process [58]. SIRT1 inhibition reduced PGC-1α expression and promoted neuronal damage in the hippocampal region of Sprague-Dawley rats [59]. Reduced SIRT1 levels were also associated with enhanced oxidative stress and caspase-3 expression, which are prominent modulators of neuronal apoptosis [59]. Therefore, SIRT1 enhancement reverses these pro-apoptotic effects. Few studies report the SIRT1 mediated deacetylation of PGC-1α negate T-2 toxin induced mitochondrial dysfunctioning and improve the state of oxidative stress in cultured human cell lines [60]. It was found that SIRT1 activator resveratrol activated AMPK pathway and enhanced the deacetylation of PGC-1α, leading to the promotion of mitochondrial biogenesis in mice [61]. In this context, it can be concluded that SIRT1 activity holds great therapeutic potential in neurodegeneration management through regulation of mitochondrial health and subsequent maintenance of energy balance in the neuronal circuit (Fig. **2**).

### 4.5 SIRT1 Reduces Neuroinflammation

Mitochondrial dysfunctioning elevates the concentration of reactive oxygen species (ROS) and upregulates nuclear factor-kappa B (NF-κB) activity that accelerates cellular deterioration [62]. Increased ROS concentration promotes neuroinflammation by evoking inflammatory responses. SIRT1 acts as an energy sensor and plays a significant role in mitochondrial health, thereby exhibiting substantial potential in negating neuroinflammation (Fig. **2**). SIRT1 activates the AMPK pathway, which is responsible for the regulation of inflammatory responses [63]. In fact, AMPK facilitates for the induction of anti-inflammatory environment, thereby reducing neuroinflammation [64]. SIRT1 mediated AMPK activation has recently been shown to inhibit neuroinflammation in BV2 microglia [65]. Suppression of NF-κB activity is another effective method of SIRT1 dependent inhibition of neuroinflammation. Enhanced NF-κB escalates ROS production. SIRT1 mediated deacetylation of the p65 subunit of NF-κB reduces neuroinflammation [66]. A recent study on male albino rats showed inhibition of neuroinflammation through SIRT1 modulated downregulation of NF-κB activity [67]. In another recent study, BML-111, a potential SIRT1 activator, ameliorated the state of neuroinflammation in mice through SIRT1/NF-κB signaling pathway [68]. The effects were reversed by EX527, a SIRT1 inhibitor. The study also showed that SIRT1 activation reduced cognitive impairment in mice. Behavioral defects were also improved by resveratrol in neonatal hypoxic-ischemic brain injury (HIBI) [69]. Resveratrol reduced the induced inflammation of neurons and subsequently diminished the microglial activation. Primary neuronal damage was found to be reduced and active involvement of NF-κB signaling pathway was reported. Positive role of SIRT1 was confirmed as the anti-inflammatory and neuroprotective effects were reduced by SIRT1 inhibition through EX527 [69]. SIRT1 activation through 17β-Estradiol in rat brain exhibited decreased ROS production and inhibited p53 acetylation [70]. NF-κB activity was found to be reduced in BV2 microglial cells. The anti-inflammatory and neuroprotective response progressed through SIRT1/p53 pathway. Such findings earmark SIRT1 as a vital target for inducing anti-inflammatory and neuroprotective responses.

## 5 ROLE OF SIRT1 IN NEURODEGENERATIVE DISORDERS

Discussion in previous sections has shed light on the role played by SIRT1 in ameliorating and preventing the progression of pathological conditions that promote neurodegeneration. Genetic and pharmacological modulation of SIRT1 has shown excellent therapeutic potency in a myriad of neuropathological conditions. Different neurodegenerative disorders present a variety of neurological conditions that demand a unique and specific response for the suppression of symptoms and subsequent inhibition of progression. This makes it imperative to discuss the role of SIRT1 in the different neurological disorders that are most commonly encountered.

### 5.1 Alzheimer’s Disease (AD)

Increased life expectancy has made Alzheimer’s disease (AD) one of the most prominent neurological anomalies in aged individuals [71]. AD is characterized by the localization of misfolded proteins in the CNS as amyloid β (Aβ) peptides. The tau protein forms neurofibrillary tangles in the cell and results in abnormal aggregation. These toxic misfolded proteins result in enhanced oxidative stress, neuroinflammation, and synaptic impairment and thereby accelerate neuronal death [72-74]. Oxidative stress and mitochondrial dysfunctioning are the major elements for the genesis and progression of AD. SIRT1 reduces ROCK1 (Rho associated coiled-coil containing protein kinase 1) expression and fosters anti-amyloidogenic cleavage of Aβ protein precursor through α-secretase, thereby inhibiting toxic protein aggregation [75]. SIRT1 is also responsible for the upregulation of basal autophagy rate that is vital for getting rid of already accumulated toxic protein aggregates [76]. This facilitates both inhibition and cleansing. AD progression is hampered by SIRT1 mediated regulation of FoxO3a expression, which is responsible for promoting p53 activity and ROCK1 gene expression. Its inhibition by SIRT1 increases protection against the toxicity of Aβ [77]. The neuroprotective pathway of inhibited FoxO3a acetylation in the neuronal space by SIRT1 activation is also a characteristic action seen in calorie restriction (CR) [69]. SIRT1 activation attenuated Aβ peptide generation in Tg2576 mice through the regulation of the ROCK1 gene [69]. Aβ concentration was found to be negatively correlated with SIRT1 activation in CR treated monkeys [70]. The therapeutic significance of such correlation is substantiated through inhibited dopaminergic neuronal loss and motor function impairment due to CR in the animal models [78].

**Fig. (3):**
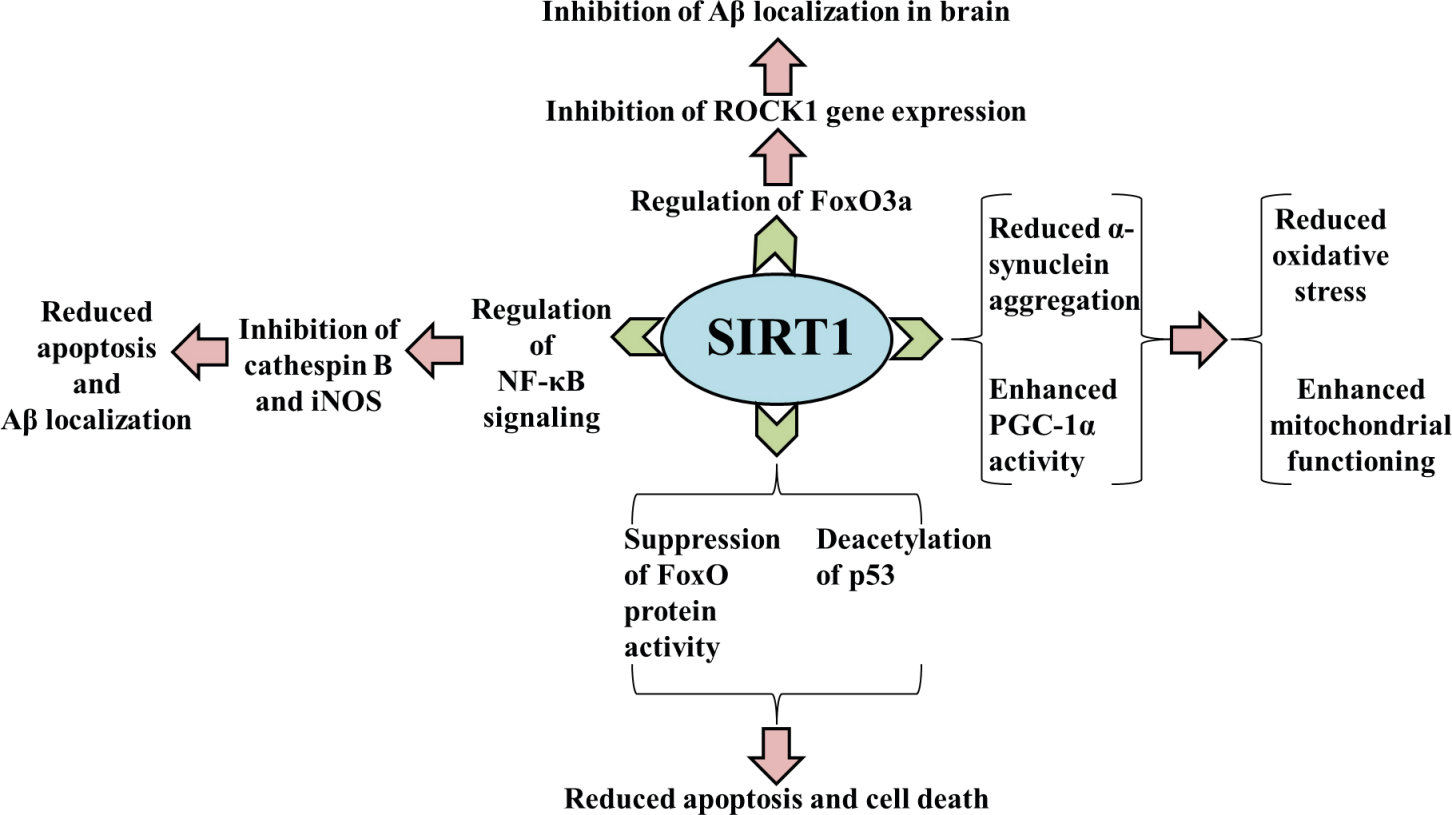
SIRT1 regulates a variety of molecular targets that have certain direct effect on neuronal health and cell survival. SIRT1 regulates FoxO3a to inhibit the expression of ROCK1 gene. Aβ localization gets inhibited due to reduced expression of ROCK1 gene and that significantly reduces neurodegeneration. SIRT1 also decreases apoptosis in cells by inhibiting the expression of cathepsin B and iNOS through regulation of NF-κB gene. This process of gene silencing eventually leads to reduced inflammation and neurodegeneration through decreased apoptosis and Aβ localization. SIRT1 is also responsible for maintaining cellular health by ensuring proper functioning. It enhances PGC1-α expression while reducing synuclein aggregation. This reduces oxidative stress in the brain and helps in the proper functioning of mitochondria. Deacetylation of p53 and suppression of FoxO protein mediated through increased SIRT1 expression also reduces cellular loss in the brain.

**Fig. (4):**
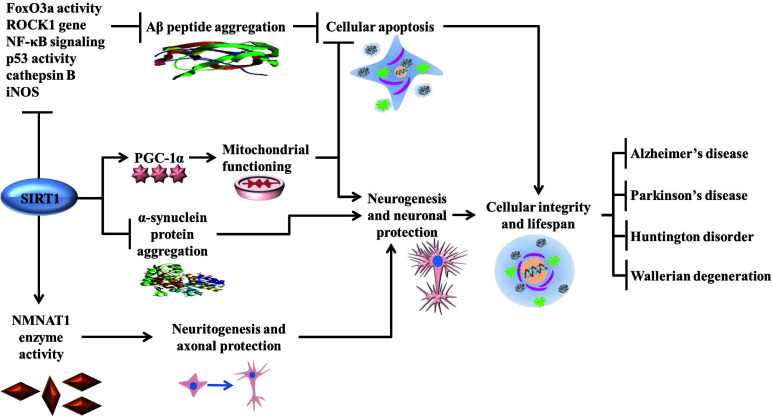
The figure depicts the central role of SIRT1 as a regulatory molecule in various neurological disorders, namely Alzheimer’s disease, Parkinson’s disease, Huntington’s disease, and Wallerian degeneration, as discussed in the review. SIRT 1 regulates the expression of associated proteins and enzymes *via* inhibition or promotion mechanism. SIRT1 is also known to affect neurophysiological mechanisms by regulating the enzyme action. The positive and negative signs indicate SIRT1 mediated promoter and inhibitor activities, respectively.

Aβ toxicity in AD is also modulated through NF-κB signaling deactivation. NF-κB signaling activates Cathepsin B and nitric oxide synthase (iNOS), thereby promoting Aβ peptide formation, leading eventually to apoptosis, neuronal death, and neurodegeneration [79-81]. SIRT1 suppresses the transcriptional activity of NF-κB that inhibits the localization of Aβ plaques and prevents neuronal death and brain atrophy [82-85]. Reduced NF-κB expression in the microglial space inhibited toxic protein aggregation and prevented the progression of Aβ toxicity [86]. SIRT1 activation by resveratrol in transgenic mouse models of AD showed reduced Aβ plaque formation [87]. The protective action was found to progress through SIRT1/AMPK pathway activation. These findings suggest that SIRT1 can be an excellent therapeutic target in AD management [71]. It exhibits significant potential in negating pathological symptoms and inhibiting cellular and molecular processes that promote AD progression (Figs. **3** and **4**). However, more research is needed to authenticate the efficacy of SIRT1 activators in AD treatment. Patients with AD have been known to suffer from a late onset disorder, which is not the consequence of a similar mutation in the amyloid precursor protein that is responsible for AD progression and tau pathology. Thus, apart from ongoing clinical trials, more work needs to be done for developing specific SIRT1 activators.

### 5.2 Parkinson’s Disease (PD)

PD is a chronic and progressive nervous system disorder that is caused as a result of the loss of dopaminergic neurons in the substantia nigra of the brain [88]. PD affects almost 2% of the population that has crossed 65 years of age. Various clinical features including rigidity, bradykinesia, postural loss, instability, and resting tremors characterize it. The α-synuclein protein plays the major role in PD pathogenesis. It gets degraded due to the process of autophagy that is regulated by SIRT1 [89, 90]. It has been found in the study performed on patients suffering from PD that various heterozygous sequence variants may change the site of transcription factor for SIRT1 promoter, thereby diminishing the SIRT1 expression [91]. A decrease in the SIRT1 level increases the risk of PD [91].

The interaction of SIRT1 and PD involves various mechanisms (Fig. **4**). Animal studies have shown that α-synuclein aggregation is reduced due to the overexpression of SIRT1 [92] (Fig. **3**). SIRT1 safeguarded human neuroblastoma cell line (SK-N-BE cells) against α-synuclein induced toxicity and eventually oxidative stress, signifying its neuroprotective effect against α-synuclein induced neurotoxicity [93]. Reduced SIRT1 expression due to the introduction of inhibitors like sirtinol can reverse the neuroprotective action of SIRT1 [94]. On the other hand, SIRT1 promoters and activators like resveratrol have been found to decrease the rate of neuronal death in both *in vivo* and *in vitro* PD models that indicate the role of SIRT1 as a major neuroprotector [95, 96].

Mitochondrial dysfunctioning is another important parameter that governs the genesis and progression of PD [97-99]. Hindrance in the mitochondrial electron transport complex activity due to various toxins is a cause of PD [100]. Other culprits for PD associated with mitochondrial function involve attenuation of mitochondrial movement and undesired enhancement of mitochondrial permeability and transition NOS activity [101, 102]. Extensive research involving mitochondrial toxins like 3NPA, MPP, and rotenone have shown that these toxins lower the SIRT1 expression in primary cultures that signifies a loss of neuroprotection against oxidative stress and mitochondrial dysfunctioning caused by these toxins [103]. SIRT1 activators impart the necessary neuroprotection by ensuring normal mitochondrial functioning and reduced oxidative stress. SIRT1 activation upregulates PGC1α, which in turn protects dopaminergic neurons against MPTP-induced cellular degeneration [104]. Enhanced SIRT1 activation and subsequent mitochondrial biogenesis regulate autophagy and mitophagy that have been previously associated with diminished α-synuclein toxicity [105] and consequently reduced PD progression.

### 5.3 Huntington’s Disease (HD)

Huntington’s disease (HD) is dominantly hereditary and progressive and is characterized by cognitive impairment, difficulty in movement, and premature death. HD is usually found in conjunction with several other psychiatric abnormalities like irritability and apathy. At the molecular level, HD is characterized by unusual protein aggregation, damage to neurons in the region of the basal ganglia, and, ultimately neuronal death [106]. Huntingtin gene plays an important role in disease development. In fact, mutation of the huntingtin gene is responsible for HD [107]. This mutation of the huntingtin gene causes glutamine residual levels to rise at the huntingtin N terminus [107]. The mutant huntingtin is known to be toxic and fatal for the cells and causes neuronal death [108]. The mutant huntingtin gene leads to neuronal death and cell loss through a mechanism mediated *via* mitochondrial dysfunctioning [109]. The mutant huntingtin gene lowers the PGC-1α expression that eventually distorts mitochondrial functioning [109].

Sirtuins ensure proper mitochondrial functioning and thus protect the brain. One of the ways to positively regulate mitochondrial functioning is through PGC-1α because it is an indicator and regulator of mitochondrial number and function. SIRT1 deacetylates and activates PGC-1α, thus ensuring the proper functioning of the mitochondria [110]. Transgenic mice models of HD have shown that downregulation of PGC-1α imparts motor abnormalities and loss of striatal neurons, while the upregulation of PGC-1α imparts neuroprotection by saving the striatal neurons against the toxicity induced by the mutant huntingtin gene [109]. Other previously discussed pathways have also been shown to regulate SIRT1 mediated inhibition of HD progression. Upregulation of BDNF expression through SIRT1 activation inhibited HD progression in a genetic mouse model [111]. FoxO3a deacetylation through SIRT1 enhancement has also been shown to promote cellular survival and neuronal protection in the HD models [112]. SIRT1 activator resveratrol protected neurons against HD progression in *C. elegans* models [113]. On the other hand, SIRT1 inhibition in *Drosophila* models successfully negated HD pathogenesis [114]. However, literature based evidence regarding the efficacy of SIRT1 inhibition against HD progression is sparse. Thus, more experiments are needed to enhance the clarity regarding the target and mechanism of SIRT1 mediated HD prevention and inhibition. However, these findings give a clear indication that SIRT1 regulation has a vital role to play in the retardation of HD progression (Fig. **4**).

### 5.4 Wallerian Neurodegenerative Disorder

Wallerian neurodegeneration is characterized by loss of axons after a focal insult that is followed with the breakdown of the myelin sheath [110]. Wld^S^ mutation causes the overexpression of the mutant Wld^S^ chimeric protein that eventually forms the mechanistic base for delayed regeneration. Wld^S^ comprises N-terminal 70 amino acids that are associated with ubiquitin fusion degradation protein 2a, which is fused with the nicotinamide mononucleotide adenylyltransferase 1 (NMNAT-1) sequence which is a key enzyme in the pathway of NAD biosynthesis [115]. NMNAT-1 activity is significant for the nervous system as it is suggested to impart protection against neurodegeneration [116]. Studies on mice models have shown that NMNAT-1 activity provides protection against axonal injury through the deacetylation potential of SIRT1 (Fig. **4**) [117]. Studies involving SIRT1 activators like resveratrol have shown that neuronal insult and progression of Wallerian degeneration are hindered due to SIRT1 activation [118]. Resveratrol induced SIRT1 activation in dorsal root ganglia cultures showed inhibited Wallerian degeneration progression [113]. SIRT1 mediated axonal protection was reversed due to SIRT1 inhibition that signified the neuroprotective role of SIRT1 in Wallerian degeneration [113].

### 5.5 Epilepsy

Epilepsy is a complex neurological disorder that involves a range of causative factors [119]. It is characterized by neuronal death, apoptosis, oxidative stress, and mitochondrial dysfunction. Overexpression of reactive oxygen species in the brain or inhibition of vital mitochondrial functions leads to neurological insults in the brain, thereby promoting a state of uncontrolled neuronal firing that eventually leads to epileptic seizures. These seizure episodes are characterized by postural loss, uncontrollable movements, and eventual loss of consciousness. Epileptic seizures are a hyper-synchronous state of the brain where an indefinite number of neurons send signals simultaneously. This hyperactive state of the brain can be attributed to the loss of ability of ion channels to close the circuit in time that provides a path of least resistance to the neuronal signals. Sodium ion channels are the most active channels that are involved in epileptic seizures. It is one of the most sought targets in conventional anti-epileptic drugs (AEDs). Mitochondrial dysfunction is also known to play a vital role in the progression of epileptic seizures.

SIRT1 plays a significant role in the neuropathology of epileptic seizures owing to its ability to deacetylase histones. SIRT1 thus can regulate many cell processes that include autophagy, apoptosis, and mitochondrial functions. SIRT1 ensures the integrity of mitochondrial functioning through activation of PGC1- α that activates ROS detoxifying enzymes [120]. SIRT1 was found to activate PGC1- α in a status epilepticus rat model [121]. In the same study, SIRT1 inhibition blocked PGC1- α enhancement and subsequently increased superoxide dismutase (SOD). This signified an impaired anti-oxidant defense mechanism following SIRT1 inhibition. Increased oxidative stress load promotes neuroinflammation and mitochondrial damage. Thus, a positive role of SIRT1 activation was highlighted in the study.

**Fig. (5):**
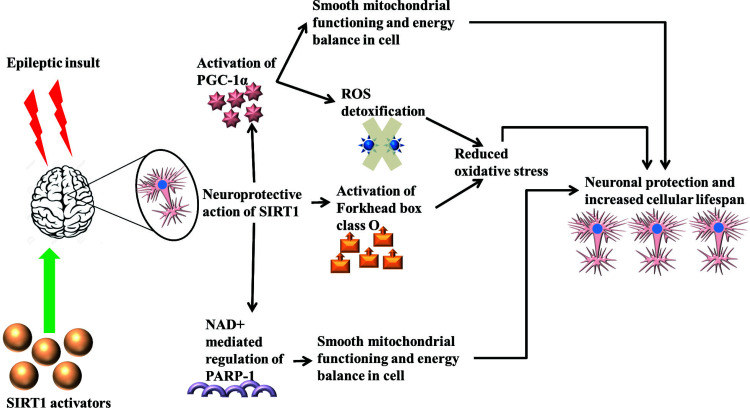
Epilepsy is characterized by increased oxidative stress in the brain. Increased concentration of ROS hampers the energy balance in the brain and thereby leads to mitochondrial dysfunctioning. SIRT1 mediated regulation of PARP1 through NAD^+^ ensures smooth mitochondrial functioning. SIRT1 promotes detoxification of ROS through detoxifying enzymes that are activated due to the activation of PGC-1α. ROS detoxification is also enhanced due to SIRT1 mediated activation of Forkhead box class O. Reduced ROS concentration promotes intact mitochondrial integrity. Activation of PGC-1α also augments mitochondrial biogenesis, which is vital for cellular survival.

SIRT1 relieves the oxidative stress from the cell also through the activation of forkhead box class O [122]. Other mechanisms through which SIRT1 protects the cells from fatal toxicity include negative regulation of p53 and NF-κB subunit p65/RelA that are actively responsible for cell mortality [123]. SIRT1 is also known to regulate DNA methyltransferase 1 activity that eventually imparts transcriptional repression, which is responsible for cell survival. SIRT1 prevents cell death due to energy failure as it regulates poly (ADP-ribose) polymerase-1 (PARP-1) through NAD^+^ [124-126] pathway [123]. Thus, SIRT1 imparts protection to the cell against epileptic seizures through various mechanisms, thereby ensuring protection against neurological abnormalities (Fig. **5**).

### 5.6 Multiple Sclerosis (MS)

**Fig. (6):**
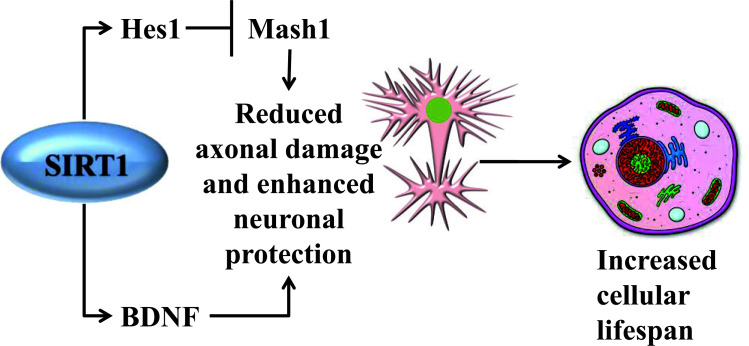
SIRT1 is a promoter of axonal health. Remyelination inhibits axonal damage and thereby enhances neuronal survival. SIRT1 mediated inhibition of Mash1 and activation of BDNF increases neuronal lifespan and thereby significantly improves coherence in the neuronal circuit. This plays an important role in various vital brain functions. Increased cellular survival and enhanced intercellular communication owing to diminished neuronal apoptosis signifies ameliorating functions of SIRT1 against neurodegeneration driven fatal molecular and biochemical alterations.

Multiple Sclerosis (MS) is a chronic CNS disorder that is characterized by inflammatory demyelination. The immune cells attack the myelin sheaths after entering the CNS thus leading to axonal disruption and substantial neuronal damage. Lesions in MS are often seen in brain, optic and other cranial nerves, and the damage caused to neurons may be permanent. Experimental autoimmune encephalomyelitis (EAE) is one of the most preferred models for the study of MS. SIRT1 activation has been shown to impart significant neuroprotection in MS by facilitating for astroglial differentiation of neural precursor cells through Hes1 binding and inhibition of Mash1 transcription (Fig. **6**) [127]. SIRT1 mediated regulation of BDNF also promotes remyelination and promotes axonal safety against neurodegenerative stimuli. Administration of SIRT1 activators, SRT501 and SRT647, inhibited the retinal ganglion cell apoptosis in SJL mice EAE model [128]. Blockage of such effect by SIRT1 inhibitor sirtinol showed that SIRT1 indeed imparts substantial neuroprotection against MS. Subsequent investigation also demonstrated that SRT501 was able to safeguard axonal density in the spinal cord [129]. Other animal models have also been reported with similar neuroprotective effects [130, 131]. SIRT1 has been seen to be significantly expressed at the chronic and acute lesion sites in individuals with MS, while no such observation was recorded for the normal brain [132]. SIRT1 does possess axonal protection potential and these vital findings emphasize its role in the subsequent inhibition of MS.

### 5.7 Spinal and Bulbar Muscle Atrophy (SBMA)

Spinal and Bulbar Muscle Atrophy (SBMA) is a rare neurological anomaly that is characterized by muscle weakening and endocrine disruption. It is a disorder specific to males and affects sensory and motor neurons. The CAG trinucleotide expansion in the amino-terminal region of the androgen receptor (AR) is known to be the cause of SBMA [133]. SIRT1 mediated deacetylation has been associated with AR repression [134]. Polyglutamine-expanded AR can also be deacetylated by SIRT1 [135]. However, these findings can only be of vital importance if the androgen receptor is a SIRT1 substrate. SIRT1 activation may have significant therapeutic potential against SBMA, but there is a need for more intensive research in this regard.

### 5.8 Amyotrophic Lateral Sclerosis (ALS)

Amyotrophic lateral sclerosis (ALS), or motor neuron disease, is characterized by the progressive loss of neurons that regulate voluntary actions through voluntary muscles. The muscular pain and stiffness in ALS culminates into decreased muscular size and compromised ability to walk, speak, and swallow [136-138]. The use of hands may also become troublesome [136]. ALS involves damage to upper motor neurons as well as lower motor neurons. While the former situation is termed as primary lateral sclerosis (PLS), the latter is called as progressive muscular atrophy (PMA) [138, 139]. Respiratory onset ALS is another variant of this disease that involves dyspnea and orthopnea [138] and may contribute greatly towards deteriorated quality of life. However, respiratory complications in ALS are rare and encountered only in about 3% of cases [138]. The onset and progression of ALS are directly dependent upon age and associated comorbidities. The progression tends to be slower in patients with age less than 40 and free from comorbidities like obesity [140, 141].

**Fig. (7):**
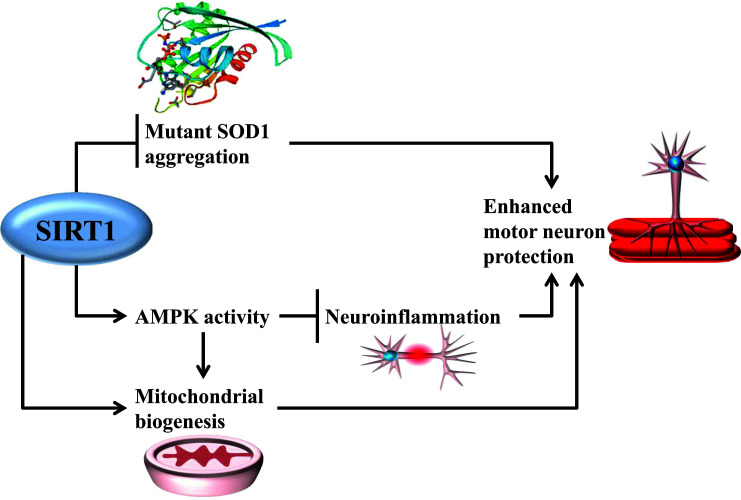
ALS is characterized by motor neuron dysfunctioning leading to hampered voluntary movement. The SOD1 protein mutations result into the accumulation of toxic protein aggregates, thereby inducing significant neurotoxicity. ALS pathology also involves downregulation of AMPK pathway that promotes neuroinflammation and subsequent motor neuron dysfunctioning. Upregulation of AMPK pathway through SIRT1 activation not only lowers the inflammatory response but also enhances mitochondrial biogenesis. Reduced toxic protein aggregates and increased energy balance in the neuronal circuit dilute the adverse effects of neurotoxic stimuli and therefore imparts significant protection against motor neuron damage.

Although the exact causative factors for the genesis and progression of ALS remain elusive, mutations of TDP-43 [139], FUS [139], and SOD1 proteins along with their toxic aggregation [142-145] and reduced AMPK activity [146] have been shown in recent studies to be the primary suspects. Mutant SOD1 protein induced neurotoxicity and AMPK pathway downregulation are the most prominently studied mechanisms of ALS progression (Fig. **7**). Mutation of SOD1 protein ruptures the mechanism of calcium homeostasis and therefore enhances the oxidative stress load in the brain that eventually triggers the chain of events responsible for progressive neuronal damage [142]. Enhanced AMPK activity is associated with reduced neuroinflammation and balanced mitochondrial functioning that are vital for keeping the concentration of reactive oxygen species in check. While regulation of AMPK activity through SIRT1 mediated pathway is well known, recent findings suggesting SIRT1 mediated control of SOD1 toxicity have substantiated the role of SIRT1 in ALS management. Reduced AMPK activity in ALS patient derived mesenchymal stem cells (MSCs) was restored through resveratrol, an active SIRT1 activating agent [146]. Resveratrol enhanced the neuro-progenitor markers in MSCs and promoted the neurite outgrowth, which advocated for the neuroprotective role of SIRT1 through AMPK activation and axonal protection [146]. SIRT1 expression was found to be reduced in the spinal cord of aged wild-type mice, which again underlined the importance of SIRT1 activation in ALS management [142]. Overexpression of SIRT1 delayed ALS progression in SOD1^G93A^ mice [142], while it attenuated the adverse effects of normal ageing on neuromuscular junctions [142]. Such ameliorating effects of overexpressed SIRT1 were also seen in SOD1^G37R^ mice [143]. Enhanced SIRT1 activity through the administration of resveratrol significantly negated the neurotoxicity induced by the mutation of SOD1 protein [143]. SIRT1 knockout has, however, been shown not to have much reverse effects in this context, which signifies a possible compensatory role of other members of the sirtuin family, thereby advocating for extensive efforts for deciphering the exact mechanism of ALS and its progression [143-145].

### 5.9 Ischemic Stroke

Stroke is one of the most prominent reasons for adult disability worldwide, with ischemic stroke contributing to more than 80% of the total cases [147]. Ischemic stress mediated occlusion of the cerebral artery causes severe deficiency of oxygen and energy that leads to the collapse of the cerebral network and ultimate neuronal death [148]. The pathological and physiological effects such as oxidative stress, neuroinflammation, and apoptosis follow the initial phase of necrosis [148]. The existing therapies against ischemic stroke have shown some extent of effectiveness; however much is still to be achieved.

SIRT1 is a potential therapeutic target against ischemic stroke as it can ameliorate the gene expression imbalance besides promoting cellular metabolism. SIRT1 exhibits anti-apoptotic and anti-inflammatory actions in ischemic stroke through the downregulation of p53 and NF-kB activity [149]. SIRT1 activation through curcumin has also been shown to diminish p53 activity and induce anti-inflammatory responses [150]. Infarction volume has also been shown previously to be regulated through SIRT1 expression. Arctigenin mediated SIRT1 activation in the middle cerebral artery occlusion (MCAO) model led to reduced infarction volume [151]. Another study employing MCAO model showed that lanthionine ketimine-5-ethyl ester (LKE) induced SIRT1 upregulation and PARP1 inhibition fortified ischemic brain tissue [152].

The protective action of SIRT1 in ischemic stroke is also regulated through the negation of oxidative stress. Elevated concentration of free radicals hampers mitochondrial functioning, thereby aggravating the ischemic insult. SIRT1 does have the ability to regulate mitochondrial functioning through PGC-1α modulation, which is vital for mitochondrial biogenesis. In this context, SIRT1 upregulation through resveratrol negated ischemic stress through PGC-1α regulation [153]. Such protective effects of SIRT1- PGC-1α signaling pathway against ischemic insult were also mimicked by Icariin (ICA) [154, 155]. Decreased mitochondrial uncoupling protein-2 (UCP2) activity and enhanced BDNF expression are other pathways mediated through SIRT1 regulation that protect neurons in ischemic stroke [156, 157]. The elevated state of oxidative stress in the ischemic insult also results in DNA damage and accumulation of DNA lesions. This imparts great importance to the DNA repair ability for ensuring neuronal protection. SIRT1 activity regulates DNA repair mechanisms and thus plays a crucial role in the therapeutic context. SIRT1 activation negates the adverse effects of PARP1 activity through replenished cellular NAD^+^, thereby facilitating for DNA repair against ischemic insult induced cellular damage [158, 159]. Resveratrol induced SIRT1 activation has been shown to downregulate PARP1 activity and attenuate DNA damage [160], thereby underlining the role of SIRT1 against ischemic stroke.

**Fig. (8):**
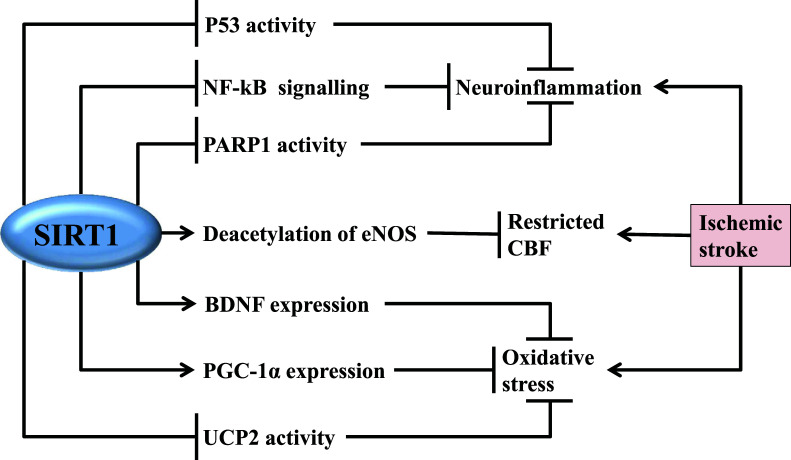
Ischemic stroke is characterized by restricted cerebral blood flow (CBF) that leads to deficiency of oxygen and energy in the cerebral tissues. Neuronal damage following restricted CBF is augmented by enhanced neuroinflammation and oxidative stress load. SIRT1 regulates various pathways that are responsible for attenuating responses aggravating the ischemic insult. SIRT1 upregulation checks p53 and PARP1 activity and diminishes NF-kB signaling, thereby negatively regulating the pro-inflammatory environment. The deacetylation of eNOS by SIRT1 inhibits the restriction of CBF and thus prevents neuronal damage in cerebral tissues due to oxygen and energy deficiency. SIRT1 maintains the energy balance in the cellular environment also through the regulation of free radical concentration. SIRT1 mediated enhancement of BDNF and PGC-1α expression and inhibition of UCP2 activity augments mitochondrial biogenesis and thereby reduces oxidative stress load. Such protective effects of SIRT1 make it an effective therapeutic target in ischemic stroke management.

Another important aspect of ischemic injury is the restricted cerebral blood flow (CBF) that causes severe deprivation of oxygen and energy in the cerebral tissues. SIRT1 mediated deacetylation of endothelial nitric oxide synthase (eNOS) promotes balanced CBF [161] and therefore prevents tissue damage and neuronal death. The Sirt1-eNOS-NO system enhances vascular relaxation and prevents the compromise of the blood brain barrier [161]. These effects are important for promoting CBF. The above findings do indicate an ameliorative effect of SIRT1 regulation in ischemic stroke through various mechanisms (Fig. **8**) and thus project it as an effective therapeutic target.

## CONCLUSION AND PROSPECTS

Aging is one of the most prominent risk factors responsible for various neurological abnormalities. The molecular changes that trigger the neurological imbalance leading to neurological disorders become more prominent as age increases [162-164]. SIRT1 has been shown to exhibit excellent neuroprotective properties in animal and human studies owing to its ability to negate pro-oxidative stimuli and induce anti-inflammatory responses within the neuronal *milieu*. Literature based evidence proves its role in the promotion of neurogenesis, mitochondrial biogenesis, and balanced energy metabolism. Many co-morbid conditions like diabetes, obesity and hypertension further deteriorate the aging process [162, 164] and different mechanisms [165] rescuing different cells get benefitted by the action of SIRT1. Its role against various neurodegenerative stimuli is substantiated by the neuroprotective actions exhibited by various SIRT1 regulating agents (Table **1**). SIRT1 modulators like resveratrol, epigallocatechin-3-gallate, and quercetin activate the SIRT1/AMPK pathway, thereby negating neuroinflammatory responses. Anti-inflammatory response is also enhanced by BML-111 and 17β-Estradiol through the silencing of NF-κB signaling pathway and downregulation of p53 activity. Such anti-inflammatory actions are also mimicked by curcumin, which also inhibits p53 activity and thereby reduces cellular apoptosis. SIRT1 activation through SRT501 and SRT647 administration has also been associated with lowered cellular apoptosis. The neuroprotective action of SIRT1 modulators also involves an ameliorated state of oxidative stress. Proper mitochondrial functioning facilitates a strengthened free radical scavenging mechanism thereby attenuating other effects such as neuroinflammation and DNA damage. Resveratrol, icariin, and epigallocatechin-3-gallate promote the activation of SIRT1/ PGC-1α pathway that is vital for mitochondrial health. Resveratrol also facilitates an improved DNA repair mechanisms and thus proves the role of SIRT1 regulation in the improvement of cellular lifespan. Inhibition of neurodegeneration against ischemic insult through SIRT1 activation by mangiferin also exemplifies the active role played by SIRT1 against progressive neuronal loss. Cellular lifespan is also increased by the SIRT1 regulators through the process of neuritogenesis. Resveratrol, SRT501, and SRT647 are known to enhance neurite outgrowth that eventually increases axonal life and promotes neuronal fortification. A decrease in neuronal loss is also achieved through gene silencing and inhibition of mutant protein induced neurotoxicity. While resveratrol and Lanthionine ketimine-5-ethyl ester (LKE) inhibit PARP1 activity, SIRT1 activator rutin downregulates NF-kB signaling pathway and thereby prevents mitochondrial dysfunctioning and subsequent neuroinflammation. Pro-inflammatory response and elevated oxidative stress are also encountered due to restricted cerebral blood flow. In this context, SIRT1 activator arctigenin promotes a reduction in infarction volume and thus alleviates pro-inflammatory responses and oxidative stress. Hence, it is clear that SIRT1 modulating agents show significant capabilities against neurodegenerative stimuli and thus project SIRT1 as an exciting therapeutic target.

However, there are issues that need to be addressed through extensive research. The development of new therapeutic strategies needs to consider the safety of human subjects with utmost urgency. SIRT1 overexpression may result in over consumption of NAD^+^, which may be detrimental for cells. The role of energy depletion in the progression of neurodegenerative disorders is well known. Over consumption of NAD^+^ and subsequent depletion of cellular energy may render neurons vulnerable to excitotoxicity. Thus, the positive effects of SIRT1 activation would strongly depend upon the level of SIRT1 activity and the prevailing energy status of the cell. Hence, this aspect needs to be investigated in animal models properly to facilitate positive alterations.

## Figures and Tables

**Table 1:** SIRT1 regulating drugs/agents and their respective actions against neurodegenerative stimuli.

**S. No.**	**SIRT1 Regulating Drugs/Agents**	**Action**	**References**
1	BML-111	Upregulation of SIRT1 activity and downregulation of NF-κB signaling pathway leading to reduced neuroinflammation	[68]
2	17β-Estradiol	SIRT1 activation, inhibition of p53 activity and downregulation of NF-κB signaling pathway thereby reducing ROS concentration and neuroinflammation	[70]
3	Resveratrol	Activation of SIRT1/AMPK pathway, inhibition of toxic protein aggregation, neuronal fortification in *in vivo* and *in vitro* models through gene silencing and enhanced axonal protection mediated through increased neurite outgrowth	[87, 95, 96, 113, 118]
4	SRT501 and SRT647	Attenuation of cellular apoptosis through SIRT1 activation and promotion of neurite outgrowth leading to increased axonal density	[128, 129]
5	Resveratrol	Increased neuro-progenitor markers and inhibition of protein mutation induced neurotoxicity	[143, 146]
6	Curcumin	SIRT1 activation, inhibition of p53 activity and enhancement in pro-inflammatory responses	[150]
7	Arctigenin	SIRT1 activation and reduced infarction volume in middle cerebral artery occlusion model	[151]
8	Lanthionine ketimine-5-ethyl ester (LKE)	SIRT1 upregulaton and PARP1 inhibition	[152]
9	Resveratrol	Upregulation of SIRT1/PGC-1α signaling pathway and enhanced mitochondrial biogenesis	[153]
10	Icariin (ICA)	SIRT1 mediated enhancement in PGC-1α expression and subsequent promotion of mitochondrial functioning	[154, 155]
11	Resveratrol	Downregulation of PARP1 activity and consequent attenuation of DNA damage	[160]
12	Rutin	SIRT1 activation and downregulation of NF-kB signaling pathway	[166]
13	Mangiferin	SIRT1 upregulation and inhibited neurodegenration in ischemic insult	[167]
14	Epigallocatechin-3-Gallate	Activation of SIRT1/AMPK and SIRT1/ PGC-1α pathway	[168]
15	Quercetin	Upregulation of SIRT1/AMPK pathway	[169]

## LIST OF ABBREVIATIONS

α-MSH: α-Melanocyte-Stimulating Hormone
A β: Amyloid β
AD: Alzheimer’s Disease
AEDs: Anti-Epileptic Drugs
AgRP: Agouti-Related Peptide
ALS: Amyotrophic Lateral Sclerosis
AMPK: Adenosine Monophosphate-Activated Protein Kinase
AR: Androgen Receptor
BDNF: Brain-Derived Neurotrophic Factor
CBF: Cerebral Blood Flow
CNS: Central Nervous System
CR: Calorie Restriction
EAE: Experimental Autoimmune Encephalomyelitis
eNOS: Endothelial Nitric Oxide Synthase
ERK1/2: Extracellular Signal-Regulated Kinase
FoxO1: Forkhead Box Protein O 1
GSK3: Glycogen Synthase Kinase 3
HDACs: Histone Deacetylases
HD: Huntington’s Disease
HIBI: Hypoxic-Ischemic Brain Injury
ICA: Icariin
IGF1: Insulin-like Growth Factor 1
LKE: Lanthionine Ketimine-5-ethyl Ester
MAPK: Mitogen-Activated Protein Kinase
MCAO: Middle cerebral Artery Occlusion
NMNAT1: Nicotinamide Mononucleotide Adenylyltransferase 1
MS: Multiple Sclerosis
MSCs: Mesenchymal Stem Cells
mtDNA: Mitochondrial DNA
NAD: Nicotinamide Adenine Dinucleotide
NADH: Nicotinamide Adenine Dinucleotide (NAD)^+^ Hydrogen
NF-κB: Nuclear Factor-Kappa B
NOS: Nitric Oxide Synthases
NRF: Nuclear Respiratory Factors
PARP-1: Poly (ADP-ribose) Polymerase-1
PD: Parkinson’s Disease
PGC-1α: PPARγ Coactivator-1α
PI3: Phosphoinositide 3
PLS: Primary Lateral Sclerosis
PMA: Progressive Muscular Atrophy
POMC: Pro-Opiomelanocortin
PPARγ: Proliferator-Activated Receptor Gamma
ROS: Reactive Oxygen Species
ROCK1: Rho Associated Coiled-coil Containing Protein Kinase 1
SBMA: Spinal and Bulbar Muscle Atrophy
SOD: Superoxide Dismutase
SVZ: Sub-Ventricular Zone
TFAM: Mitochondrial Transcription Factor A
UCP2: Uncoupling Protein-2
